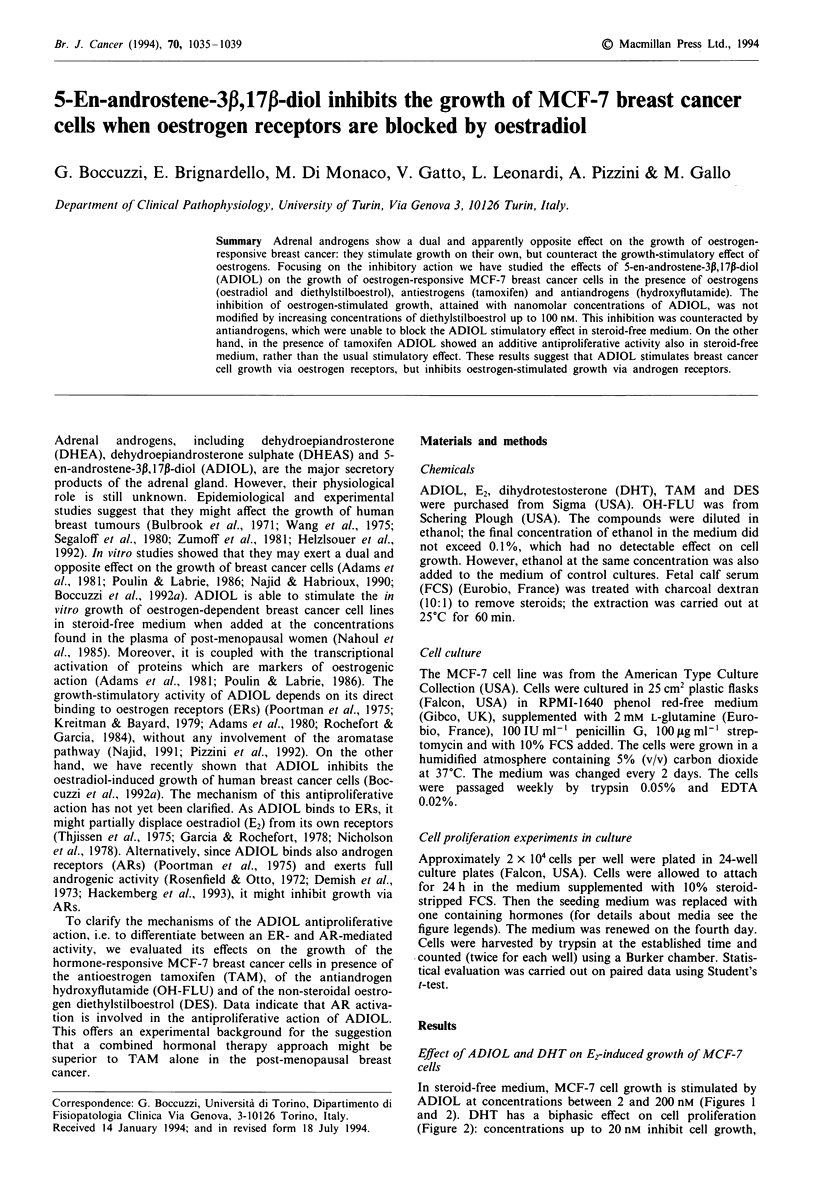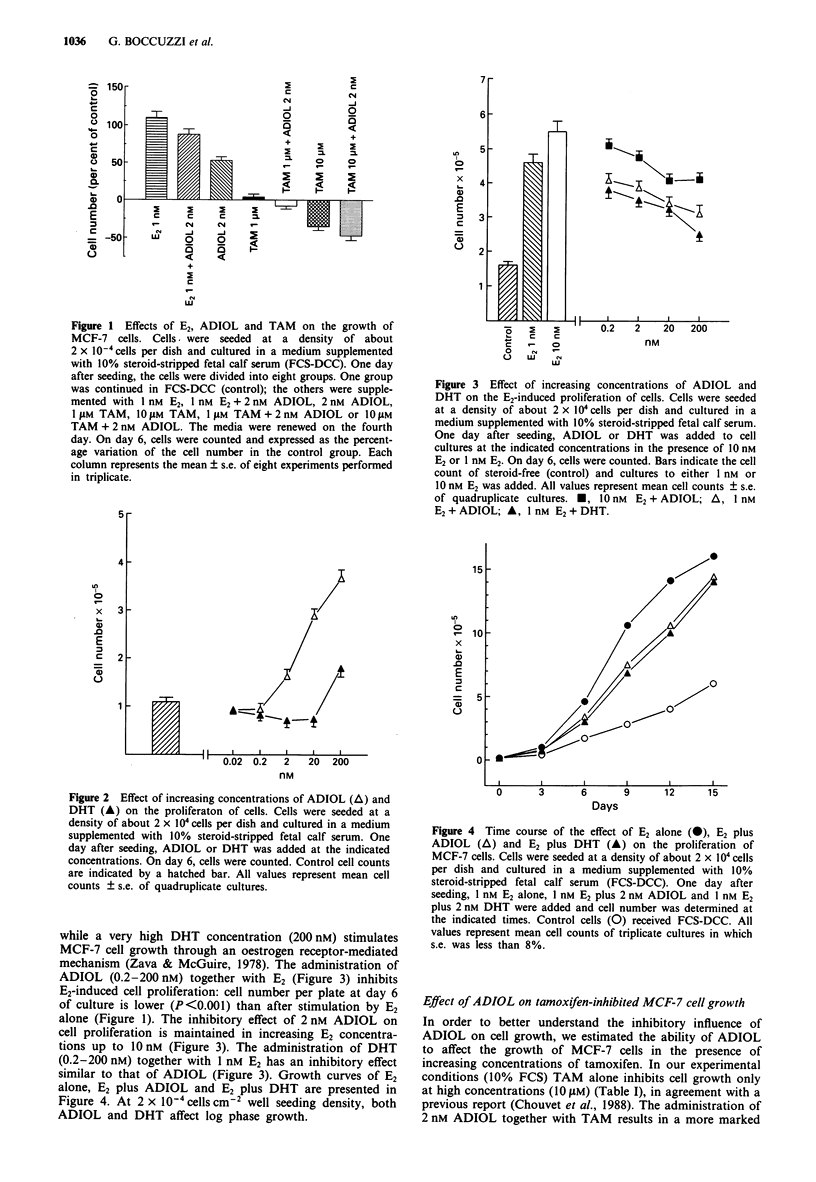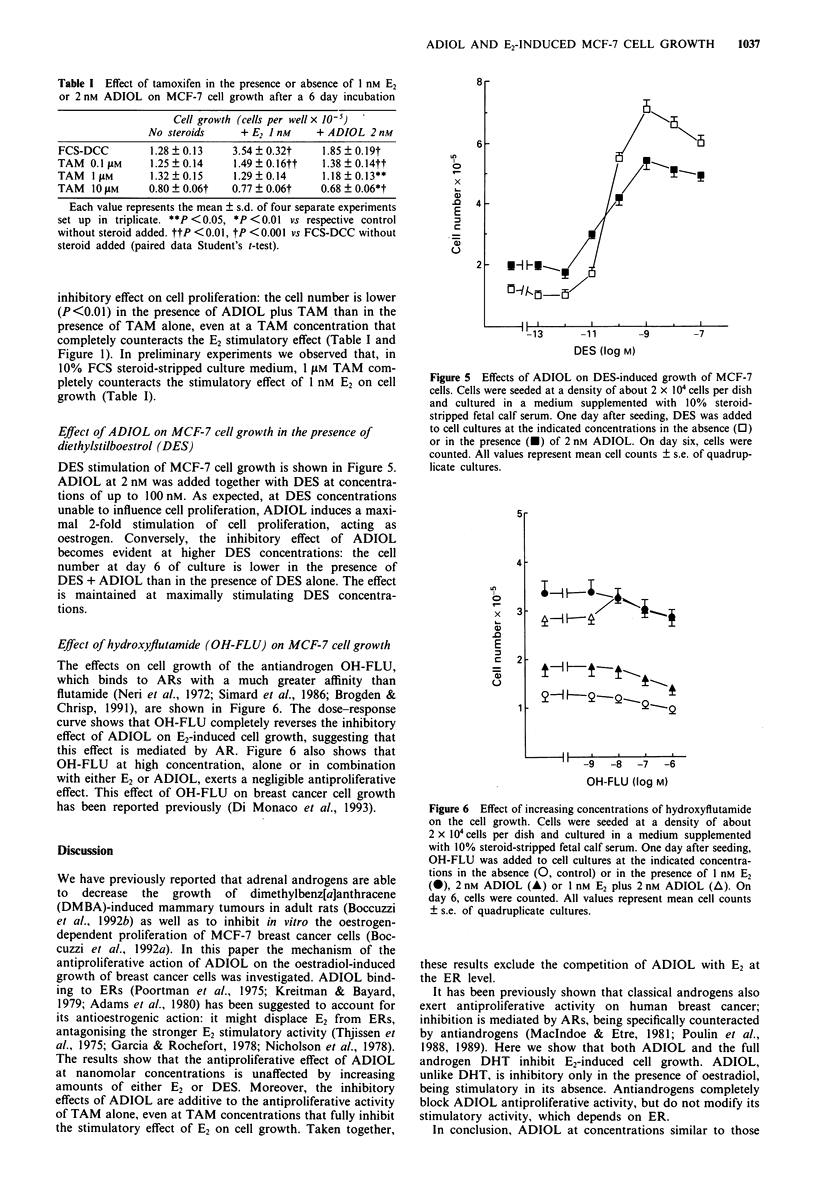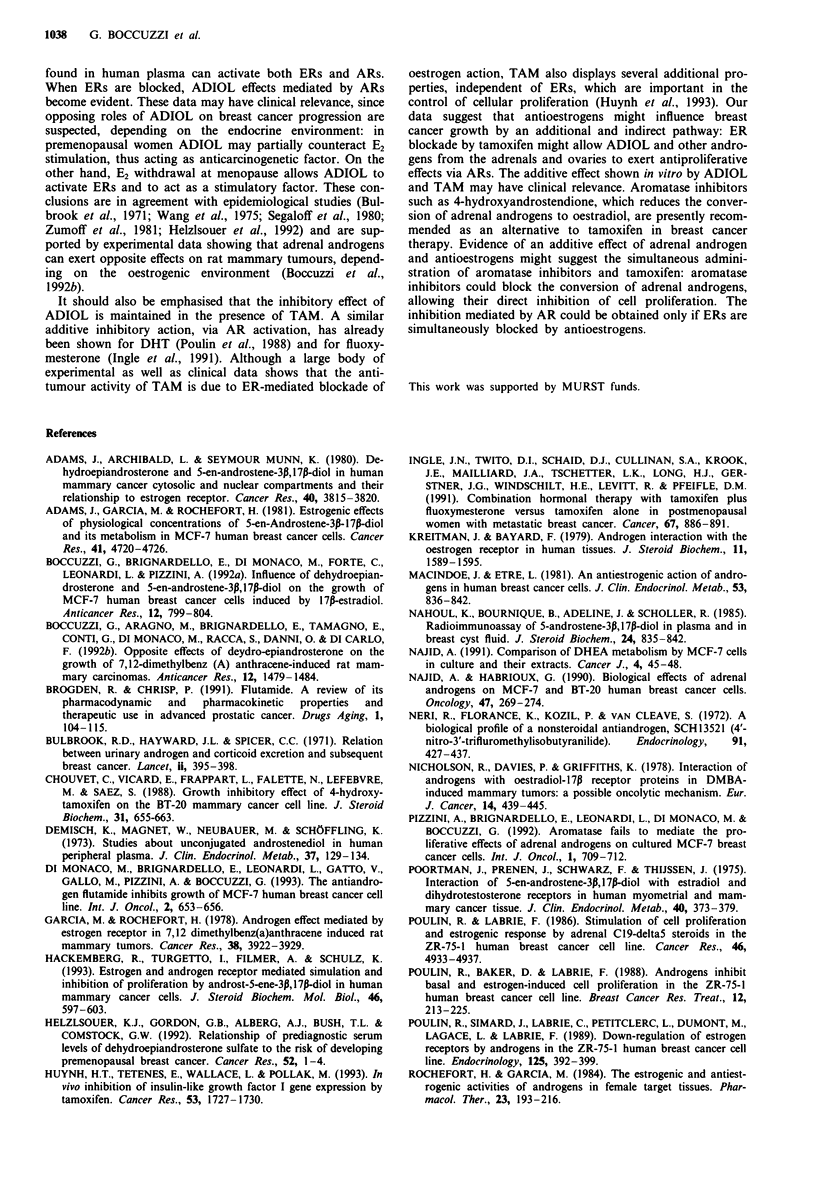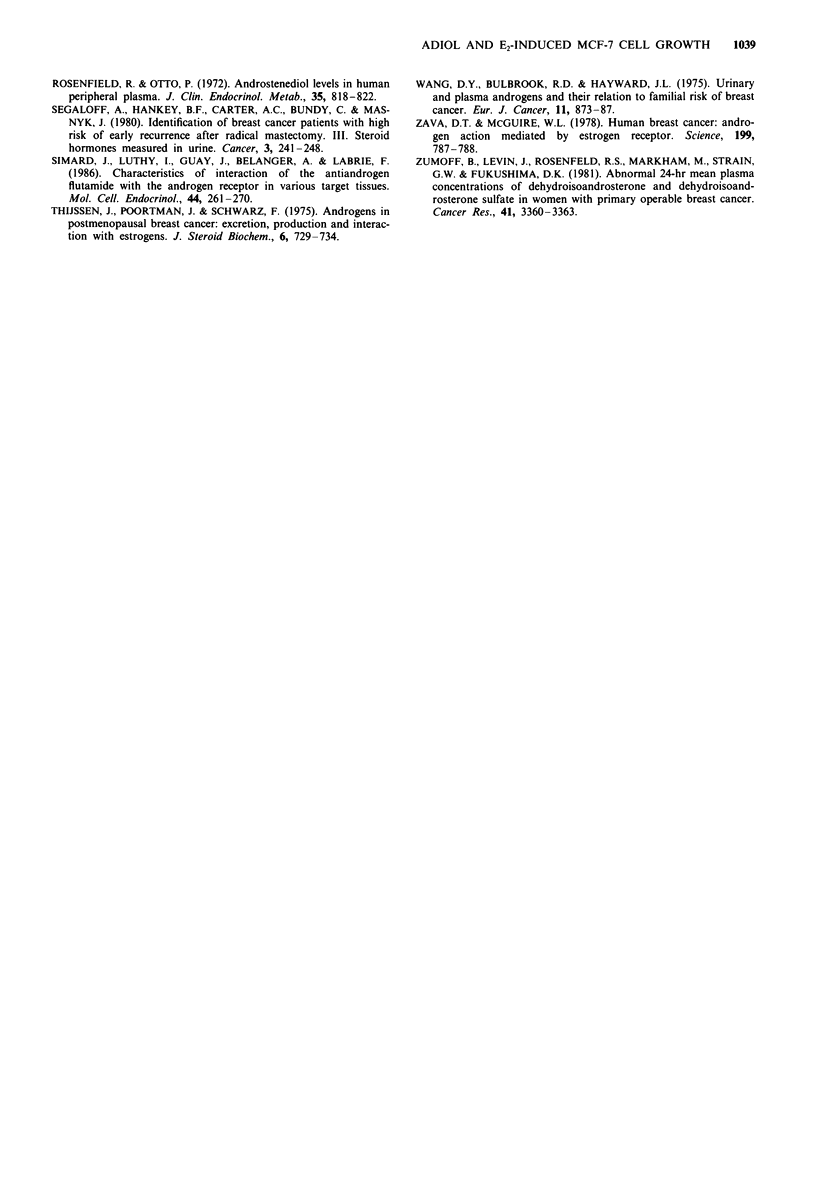# 5-En-androstene-3 beta,17 beta-diol inhibits the growth of MCF-7 breast cancer cells when oestrogen receptors are blocked by oestradiol.

**DOI:** 10.1038/bjc.1994.444

**Published:** 1994-12

**Authors:** G. Boccuzzi, E. Brignardello, M. Di Monaco, V. Gatto, L. Leonardi, A. Pizzini, M. Gallo

**Affiliations:** Department of Clinical Pathophysiology, University of Turin, Italy.

## Abstract

Adrenal androgens show a dual and apparently opposite effect on the growth of oestrogen-responsive breast cancer: they stimulate growth on their own, but counteract the growth-stimulatory effect of oestrogens. Focusing on the inhibitory action we have studied the effects of 5-en-androstene-3 beta,17 beta-diol (ADIOL) on the growth of oestrogen-responsive MCF-7 breast cancer cells in the presence of oestrogens (oestradiol and diethylstilboestrol), antiestrogens (tamoxifen) and antiandrogens (hydroxyflutamide). The inhibition of oestrogen-stimulated growth, attained with nanomolar concentrations of ADIOL, was not modified by increasing concentrations of diethylstilboestrol up to 100 nM. This inhibition was counteracted by antiandrogens, which were unable to block the ADIOL stimulatory effect in steroid-free medium. On the other hand, in the presence of tamoxifen ADIOL showed an additive antiproliferative activity also in steroid-free medium, rather than the usual stimulatory effect. These results suggest that ADIOL stimulates breast cancer cell growth via oestrogen receptors, but inhibits oestrogen-stimulated growth via androgen receptors.


Br. J. Cancer (1994), 70, 1035–1039 © Macmillan Press Ltd., 1994

### 5-En-androstene-3β,17β-diol inhibits the growth of MCF-7 breast cancer cells when oestrogen receptors are blocked by oestradiol

G. Boccuzzi, E. Brignardello, M. Di Monaco, V. Gatto, L. Leonardi, A. Pizzini & M. Gallo

*Department of Clinical Pathophysiology, University of Turin, Via Genova 3, 10126 Turin, Italy.*

**Summary** Adrenal androgens show a dual and apparently opposite effect on the growth of oestrogen-responsive breast cancer: they stimulate growth on their own, but counteract the growth-stimulatory effect of oestrogens. Focusing on the inhibitory action we have studied the effects of 5-en-androstene-3β,17β-diol (ADIOL) on the growth of oestrogen-responsive MCF-7 breast cancer cells in the presence of oestrogens (oestradiol and diethylstilboestrol), antiestrogens (tamoxifen) and antiandrogens (hydroxyflutamide). The inhibition of oestrogen-stimulated growth, attained with nanomolar concentrations of ADIOL, was not modified by increasing concentrations of diethylstilboestrol up to 100 nM. This inhibition was counteracted by antiandrogens, which were unable to block the ADIOL stimulatory effect in steroid-free medium. On the other hand, in the presence of tamoxifen ADIOL showed an additive antiproliferative activity also in steroid-free medium, rather than the usual stimulatory effect. These results suggest that ADIOL stimulates breast cancer cell growth via oestrogen receptors, but inhibits oestrogen-stimulated growth via androgen receptors.

Adrenal androgens, including dehydroepiandrosterone (DHEA), dehydroepiandrosterone sulphate (DHEAS) and 5-en-androstene-3β,17β-diol (ADIOL), are the major secretory products of the adrenal gland. However, their physiological role is still unknown. Epidemiological and experimental studies suggest that they might affect the growth of human breast tumours (Bulbrook *et al.*, 1971; Wang *et al.*, 1975; Segaloff *et al.*, 1980; Zumoff *et al.*, 1981; Helzlsouer *et al.*, 1992). *In vitro* studies showed that they may exert a dual and opposite effect on the growth of breast cancer cells (Adams *et al.*, 1981; Poulin & Labrie, 1986; Najid & Habrioux, 1990; Boccuzzi *et al.*, 1992*a*). ADIOL is able to stimulate the *in vitro* growth of oestrogen-dependent breast cancer cell lines in steroid-free medium when added at the concentrations found in the plasma of post-menopausal women (Nahoul *et al.*, 1985). Moreover, it is coupled with the transcriptional activation of proteins which are markers of oestrogenic action (Adams *et al.*, 1981; Poulin & Labrie, 1986). The growth-stimulatory activity of ADIOL depends on its direct binding to oestrogen receptors (ERs) (Poortman *et al.*, 1975; Kreitman & Bayard, 1979; Adams *et al.*, 1980; Rochefort & Garcia, 1984), without any involvement of the aromatase pathway (Najid, 1991; Pizzini *et al.*, 1992). On the other hand, we have recently shown that ADIOL inhibits the oestradiol-induced growth of human breast cancer cells (Boccuzzi *et al.*, 1992*a*). The mechanism of this antiproliferative action has not yet been clarified. As ADIOL binds to ERs, it might partially displace oestradiol ($E_2$) from its own receptors (Thjissen *et al.*, 1975; Garcia & Rochefort, 1978; Nicholson *et al.*, 1978). Alternatively, since ADIOL binds also androgen receptors (ARs) (Poortman *et al.*, 1975) and exerts full androgenic activity (Rosenfield & Otto, 1972; Demish *et al.*, 1973; Hackemberg *et al.*, 1993), it might inhibit growth via ARs.

To clarify the mechanisms of the ADIOL antiproliferative action, i.e. to differentiate between an ER- and AR-mediated activity, we evaluated its effects on the growth of the hormone-responsive MCF-7 breast cancer cells in presence of the antioestrogen tamoxifen (TAM), of the antiandrogen hydroxyflutamide (OH-FLU) and of the non-steroidal oestrogen diethylstilboestrol (DES). Data indicate that AR activation is involved in the antiproliferative action of ADIOL. This offers an experimental background for the suggestion that a combined hormonal therapy approach might be superior to TAM alone in the post-menopausal breast cancer.

Correspondence: G. Boccuzzi, Università di Torino, Dipartimento di Fisiopatologia Clinica Via Genova, 3-10126 Torino, Italy.
Received 14 January 1994; and in revised form 18 July 1994.

### Materials and methods

### Chemicals

ADIOL, $E_2$, dihydrotestosterone (DHT), TAM and DES were purchased from Sigma (USA). OH-FLU was from Schering Plough (USA). The compounds were diluted in ethanol; the final concentration of ethanol in the medium did not exceed 0.1%, which had no detectable effect on cell growth. However, ethanol at the same concentration was also added to the medium of control cultures. Fetal calf serum (FCS) (Eurobio, France) was treated with charcoal dextran (10:1) to remove steroids; the extraction was carried out at 25°C for 60 min.

### Cell culture

The MCF-7 cell line was from the American Type Culture Collection (USA). Cells were cultured in 25 $cm^2$ plastic flasks (Falcon, USA) in RPMI-1640 phenol red-free medium (Gibco, UK), supplemented with 2 mM L-glutamine (Eurobio, France), 100 IU $ml^{-1}$ penicillin G, 100 $\mu g\ ml^{-1}$ streptomycin and with 10% FCS added. The cells were grown in a humidified atmosphere containing 5% (v/v) carbon dioxide at 37°C. The medium was changed every 2 days. The cells were passaged weekly by trypsin 0.05% and EDTA 0.02%.

### Cell proliferation experiments in culture

Approximately $2 \times 10^4$ cells per well were plated in 24-well culture plates (Falcon, USA). Cells were allowed to attach for 24 h in the medium supplemented with 10% steroid-stripped FCS. Then the seeding medium was replaced with one containing hormones (for details about media see the figure legends). The medium was renewed on the fourth day. Cells were harvested by trypsin at the established time and counted (twice for each well) using a Burker chamber. Statistical evaluation was carried out on paired data using Student's *t*-test.

### Results

### Effect of ADIOL and DHT on $E_2$-induced growth of MCF-7 cells

In steroid-free medium, MCF-7 cell growth is stimulated by ADIOL at concentrations between 2 and 200 nM (Figures 1 and 2). DHT has a biphasic effect on cell proliferation (Figure 2): concentrations up to 20 nM inhibit cell growth,

**Figure 1** Effects of $E_2$, ADIOL and TAM on the growth of MCF-7 cells. Cells were seeded at a density of about $2 \times 10^{-4}$ cells per dish and cultured in a medium supplemented with 10% steroid-stripped fetal calf serum (FCS-DCC). One day after seeding, the cells were divided into eight groups. One group was continued in FCS-DCC (control); the others were supplemented with 1 nM $E_2$, 1 nM $E_2$ + 2 nM ADIOL, 2 nM ADIOL, 1 μM TAM, 10 μM TAM, 1 μM TAM + 2 nM ADIOL or 10 μM TAM + 2 nM ADIOL. The media were renewed on the fourth day. On day 6, cells were counted and expressed as the percentage variation of the cell number in the control group. Each column represents the mean ± s.e. of eight experiments performed in triplicate.

**Figure 2** Effect of increasing concentrations of ADIOL (Δ) and DHT (▲) on the proliferaton of cells. Cells were seeded at a density of about $2 \times 10^4$ cells per dish and cultured in a medium supplemented with 10% steroid-stripped fetal calf serum. One day after seeding, ADIOL or DHT was added at the indicated concentrations. On day 6, cells were counted. Control cell counts are indicated by a hatched bar. All values represent mean cell counts ± s.e. of quadruplicate cultures.

**Figure 3** Effect of increasing concentrations of ADIOL and DHT on the $E_2$-induced proliferation of cells. Cells were seeded at a density of about $2 \times 10^4$ cells per dish and cultured in a medium supplemented with 10% steroid-stripped fetal calf serum. One day after seeding, ADIOL or DHT was added to cell cultures at the indicated concentrations in the presence of 10 nM $E_2$ or 1 nM $E_2$. On day 6, cells were counted. Bars indicate the cell count of steroid-free (control) and cultures to either 1 nM or 10 nM $E_2$ was added. All values represent mean cell counts ± s.e. of quadruplicate cultures. ■, 10 nM $E_2$ + ADIOL; Δ, 1 nM $E_2$ + ADIOL; ▲, 1 nM $E_2$ + DHT.

**Figure 4** Time course of the effect of $E_2$ alone (●), $E_2$ plus ADIOL (Δ) and $E_2$ plus DHT (▲) on the proliferation of MCF-7 cells. Cells were seeded at a density of about $2 \times 10^4$ cells per dish and cultured in a medium supplemented with 10% steroid-stripped fetal calf serum (FCS-DCC). One day after seeding, 1 nM $E_2$ alone, 1 nM $E_2$ plus 2 nM ADIOL and 1 nM $E_2$ plus 2 nM DHT were added and cell number was determined at the indicated times. Control cells (O) received FCS-DCC. All values represent mean cell counts of triplicate cultures in which s.e. was less than 8%.

while a very high DHT concentration (200 nM) stimulates MCF-7 cell growth through an oestrogen receptor-mediated mechanism (Zava & McGuire, 1978). The administration of ADIOL (0.2–200 nM) together with $E_2$ (Figure 3) inhibits $E_2$-induced cell proliferation: cell number per plate at day 6 of culture is lower ($P<0.001$) than after stimulation by $E_2$ alone (Figure 1). The inhibitory effect of 2 nM ADIOL on cell proliferation is maintained in increasing $E_2$ concentrations up to 10 nM (Figure 3). The administration of DHT (0.2–200 nM) together with 1 nM $E_2$ has an inhibitory effect similar to that of ADIOL (Figure 3). Growth curves of $E_2$ alone, $E_2$ plus ADIOL and $E_2$ plus DHT are presented in Figure 4. At $2 \times 10^{-4}$ cells $cm^{-2}$ well seeding density, both ADIOL and DHT affect log phase growth.

### *Effect of ADIOL on tamoxifen-inhibited MCF-7 cell growth*

In order to better understand the inhibitory influence of ADIOL on cell growth, we estimated the ability of ADIOL to affect the growth of MCF-7 cells in the presence of increasing concentrations of tamoxifen. In our experimental conditions (10% FCS) TAM alone inhibits cell growth only at high concentrations (10 μM) (Table I), in agreement with a previous report (Chouvet *et al.*, 1988). The administration of 2 nM ADIOL together with TAM results in a more marked

**Table I** Effect of tamoxifen in the presence or absence of 1 nM $E_2$ or 2 nM ADIOL on MCF-7 cell growth after a 6 day incubation

| | Cell growth (cells per well $\times 10^{-5}$) | | |
|---|---|---|---|
| | No steroids | + $E_2$ 1 nM | + ADIOL 2 nM |
| FCS-DCC | 1.28 ± 0.13 | 3.54 ± 0.32† | 1.85 ± 0.19† |
| TAM 0.1 μM | 1.25 ± 0.14 | 1.49 ± 0.16†† | 1.38 ± 0.14†† |
| TAM 1 μM | 1.32 ± 0.15 | 1.29 ± 0.14 | 1.18 ± 0.13** |
| TAM 10 μM | 0.80 ± 0.06† | 0.77 ± 0.06† | 0.68 ± 0.06*† |

Each value represents the mean ± s.d. of four separate experiments set up in triplicate. **$P < 0.05$, *$P < 0.01$ *vs* respective control without steroid added. ††$P < 0.01$, †$P < 0.001$ *vs* FCS-DCC without steroid added (paired data Student's *t*-test).

inhibitory effect on cell proliferation: the cell number is lower ($P < 0.01$) in the presence of ADIOL plus TAM than in the presence of TAM alone, even at a TAM concentration that completely counteracts the $E_2$ stimulatory effect (Table I and Figure 1). In preliminary experiments we observed that, in 10% FCS steroid-stripped culture medium, 1 μM TAM completely counteracts the stimulatory effect of 1 nM $E_2$ on cell growth (Table I).

### *Effect of ADIOL on MCF-7 cell growth in the presence of diethylstilboestrol (DES)*

DES stimulation of MCF-7 cell growth is shown in Figure 5. ADIOL at 2 nM was added together with DES at concentrations of up to 100 nM. As expected, at DES concentrations unable to influence cell proliferation, ADIOL induces a maximal 2-fold stimulation of cell proliferation, acting as oestrogen. Conversely, the inhibitory effect of ADIOL becomes evident at higher DES concentrations: the cell number at day 6 of culture is lower in the presence of DES + ADIOL than in the presence of DES alone. The effect is maintained at maximally stimulating DES concentrations.

### *Effect of hydroxyflutamide (OH-FLU) on MCF-7 cell growth*

The effects on cell growth of the antiandrogen OH-FLU, which binds to ARs with a much greater affinity than flutamide (Neri *et al.*, 1972; Simard *et al.*, 1986; Brogden & Chrisp, 1991), are shown in Figure 6. The dose–response curve shows that OH-FLU completely reverses the inhibitory effect of ADIOL on $E_2$-induced cell growth, suggesting that this effect is mediated by AR. Figure 6 also shows that OH-FLU at high concentration, alone or in combination with either $E_2$ or ADIOL, exerts a negligible antiproliferative effect. This effect of OH-FLU on breast cancer cell growth has been reported previously (Di Monaco *et al.*, 1993).

### Discussion

We have previously reported that adrenal androgens are able to decrease the growth of dimethylbenz[*a*]anthracene (DMBA)-induced mammary tumours in adult rats (Boccuzzi *et al.*, 1992*b*) as well as to inhibit *in vitro* the oestrogen-dependent proliferation of MCF-7 breast cancer cells (Boccuzzi *et al.*, 1992*a*). In this paper the mechanism of the antiproliferative action of ADIOL on the oestradiol-induced growth of breast cancer cells was investigated. ADIOL binding to ERs (Poortman *et al.*, 1975; Kreitman & Bayard, 1979; Adams *et al.*, 1980) has been suggested to account for its antioestrogenic action: it might displace $E_2$ from ERs, antagonising the stronger $E_2$ stimulatory activity (Thjissen *et al.*, 1975; Garcia & Rochefort, 1978; Nicholson *et al.*, 1978). The results show that the antiproliferative effect of ADIOL at nanomolar concentrations is unaffected by increasing amounts of either $E_2$ or DES. Moreover, the inhibitory effects of ADIOL are additive to the antiproliferative activity of TAM alone, even at TAM concentrations that fully inhibit the stimulatory effect of $E_2$ on cell growth. Taken together,

**Figure 5** Effects of ADIOL on DES-induced growth of MCF-7 cells. Cells were seeded at a density of about $2 \times 10^4$ cells per dish and cultured in a medium supplemented with 10% steroid-stripped fetal calf serum. One day after seeding, DES was added to cell cultures at the indicated concentrations in the absence (□) or in the presence (■) of 2 nM ADIOL. On day six, cells were counted. All values represent mean cell counts ± s.e. of quadruplicate cultures.

**Figure 6** Effect of increasing concentrations of hydroxyflutamide on the cell growth. Cells were seeded at a density of about $2 \times 10^4$ cells per dish and cultured in a medium supplemented with 10% steroid-stripped fetal calf serum. One day after seeding, OH-FLU was added to cell cultures at the indicated concentrations in the absence (○, control) or in the presence of 1 nM $E_2$ (●), 2 nM ADIOL (▲) or 1 nM $E_2$ plus 2 nM ADIOL (△). On day 6, cells were counted. All values represent mean cell counts ± s.e. of quadruplicate cultures.

these results exclude the competition of ADIOL with $E_2$ at the ER level.

It has been previously shown that classical androgens also exert antiproliferative activity on human breast cancer; inhibition is mediated by ARs, being specifically counteracted by antiandrogens (MacIndoe & Etre, 1981; Poulin *et al.*, 1988, 1989). Here we show that both ADIOL and the full androgen DHT inhibit $E_2$-induced cell growth. ADIOL, unlike DHT, is inhibitory only in the presence of oestradiol, being stimulatory in its absence. Antiandrogens completely block ADIOL antiproliferative activity, but do not modify its stimulatory activity, which depends on ER.

In conclusion, ADIOL at concentrations similar to those found in human plasma can activate both ERs and ARs. When ERs are blocked, ADIOL effects mediated by ARs become evident. These data may have clinical relevance, since opposing roles of ADIOL on breast cancer progression are suspected, depending on the endocrine environment: in premenopausal women ADIOL may partially counteract $E_2$ stimulation, thus acting as anticarcinogenetic factor. On the other hand, $E_2$ withdrawal at menopause allows ADIOL to activate ERs and to act as a stimulatory factor. These conclusions are in agreement with epidemiological studies (Bulbrook *et al.*, 1971; Wang *et al.*, 1975; Segaloff *et al.*, 1980; Zumoff *et al.*, 1981; Helzlsouer *et al.*, 1992) and are supported by experimental data showing that adrenal androgens can exert opposite effects on rat mammary tumours, depending on the oestrogenic environment (Boccuzzi *et al.*, 1992*b*).

It should also be emphasised that the inhibitory effect of ADIOL is maintained in the presence of TAM. A similar additive inhibitory action, via AR activation, has already been shown for DHT (Poulin *et al.*, 1988) and for fluoxymesterone (Ingle *et al.*, 1991). Although a large body of experimental as well as clinical data shows that the antitumour activity of TAM is due to ER-mediated blockade of oestrogen action, TAM also displays several additional properties, independent of ERs, which are important in the control of cellular proliferation (Huynh *et al.*, 1993). Our data suggest that antioestrogens might influence breast cancer growth by an additional and indirect pathway: ER blockade by tamoxifen might allow ADIOL and other androgens from the adrenals and ovaries to exert antiproliferative effects via ARs. The additive effect shown *in vitro* by ADIOL and TAM may have clinical relevance. Aromatase inhibitors such as 4-hydroxyandrostendione, which reduces the conversion of adrenal androgens to oestradiol, are presently recommended as an alternative to tamoxifen in breast cancer therapy. Evidence of an additive effect of adrenal androgen and antioestrogens might suggest the simultaneous administration of aromatase inhibitors and tamoxifen: aromatase inhibitors could block the conversion of adrenal androgens, allowing their direct inhibition of cell proliferation. The inhibition mediated by AR could be obtained only if ERs are simultaneously blocked by antioestrogens.

This work was supported by MURST funds.

### References

ADAMS, J., ARCHIBALD, L. & SEYMOUR MUNN, K. (1980). Dehydroepiandrosterone and 5-en-androstene-3β,17β-diol in human mammary cancer cytosolic and nuclear compartments and their relationship to estrogen receptor. *Cancer Res.*, **40**, 3815–3820.

ADAMS, J., GARCIA, M. & ROCHEFORT, H. (1981). Estrogenic effects of physiological concentrations of 5-en-Androstene-3β-17β-diol and its metabolism in MCF-7 human breast cancer cells. *Cancer Res.*, **41**, 4720–4726.

BOCCUZZI, G., BRIGNARDELLO, E., DI MONACO, M., FORTE, C., LEONARDI, L. & PIZZINI, A. (1992*a*). Influence of dehydroepiandrosterone and 5-en-androstene-3β,17β-diol on the growth of MCF-7 human breast cancer cells induced by 17β-estradiol. *Anticancer Res.*, **12**, 799–804.

BOCCUZZI, G., ARAGNO, M., BRIGNARDELLO, E., TAMAGNO, E., CONTI, G., DI MONACO, M., RACCA, S., DANNI, O. & DI CARLO, F. (1992*b*). Opposite effects of deydro-epiandrosterone on the growth of 7,12-dimethylbenz (A) anthracene-induced rat mammary carcinomas. *Anticancer Res.*, **12**, 1479–1484.

BROGDEN, R. & CHRISP, P. (1991). Flutamide. A review of its pharmacodynamic and pharmacokinetic properties and therapeutic use in advanced prostatic cancer. *Drugs Aging*, **1**, 104–115.

BULBROOK, R.D., HAYWARD, J.L. & SPICER, C.C. (1971). Relation between urinary androgen and corticoid excretion and subsequent breast cancer. *Lancet*, **ii**, 395–398.

CHOUVET, C., VICARD, E., FRAPPART, L., FALETTE, N., LEFEBVRE, M. & SAEZ, S. (1988). Growth inhibitory effect of 4-hydroxytamoxifen on the BT-20 mammary cancer cell line. *J. Steroid Biochem.*, **31**, 655-663.

DEMISCH, K., MAGNET, W., NEUBAUER, M. & SCHÖFFLING, K. (1973). Studies about unconjugated androstenediol in human peripheral plasma. *J. Clin. Endocrinol. Metab.*, **37**, 129–134.

DI MONACO, M., BRIGNARDELLO, E., LEONARDI, L., GATTO, V., GALLO, M., PIZZINI, A. & BOCCUZZI, G. (1993). The antiandrogen flutamide inhibits growth of MCF-7 human breast cancer cell line. *Int. J. Oncol.*, **2**, 653–656.

GARCIA, M. & ROCHEFORT, H. (1978). Androgen effect mediated by estrogen receptor in 7,12 dimethylbenz(a)anthracene induced rat mammary tumors. *Cancer Res.*, **38**, 3922–3929.

HACKEMBERG, R., TURGETTO, I., FILMER, A. & SCHULZ, K. (1993). Estrogen and androgen receptor mediated simulation and inhibition of proliferation by androst-5-ene-3β,17β-diol in human mammary cancer cells. *J. Steroid Biochem. Mol. Biol.*, **46**, 597–603.

HELZLSOUER, K.J., GORDON, G.B., ALBERG, A.J., BUSH, T.L. & COMSTOCK, G.W. (1992). Relationship of prediagnostic serum levels of dehydroepiandrosterone sulfate to the risk of developing premenopausal breast cancer. *Cancer Res.*, **52**, 1–4.

HUYNH, H.T., TETENES, E., WALLACE, L. & POLLAK, M. (1993). *In vivo* inhibition of insulin-like growth factor I gene expression by tamoxifen. *Cancer Res.*, **53**, 1727–1730.

INGLE, J.N., TWITO, D.I., SCHAID, D.J., CULLINAN, S.A., KROOK, J.E., MAILLIARD, J.A., TSCHETTER, L.K., LONG, H.J., GERSTNER, J.G., WINDSCHILT, H.E., LEVITT, R. & PFEIFLE, D.M. (1991). Combination hormonal therapy with tamoxifen plus fluoxymesterone versus tamoxifen alone in postmenopausal women with metastatic breast cancer. *Cancer*, **67**, 886-891.

KREITMAN, J. & BAYARD, F. (1979). Androgen interaction with the oestrogen receptor in human tissues. *J. Steroid Biochem.*, **11**, 1589–1595.

MACINDOE, J. & ETRE, L. (1981). An antiestrogenic action of androgens in human breast cancer cells. *J. Clin. Endocrinol. Metab.*, **53**, 836–842.

NAHOUL, K., BOURNIQUE, B., ADELINE, J. & SCHOLLER, R. (1985). Radioimmunoassay of 5-androstene-3β,17β-diol in plasma and in breast cyst fluid. *J. Steroid Biochem.*, **24**, 835–842.

NAJID, A. (1991). Comparison of DHEA metabolism by MCF-7 cells in culture and their extracts. *Cancer J.*, **4**, 45–48.

NAJID, A. & HABRIOUX, G. (1990). Biological effects of adrenal androgens on MCF-7 and BT-20 human breast cancer cells. *Oncology*, **47**, 269–274.

NERI, R., FLORANCE, K., KOZIL, P. & VAN CLEAVE, S. (1972). A biological profile of a nonsteroidal antiandrogen, SCH13521 (4'-nitro-3'-trifluromethylisobutyranilide). *Endocrinology*, **91**, 427–437.

NICHOLSON, R., DAVIES, P. & GRIFFITHS, K. (1978). Interaction of androgens with oestradiol-17β receptor proteins in DMBA-induced mammary tumors: a possible oncolytic mechanism. *Eur. J. Cancer*, **14**, 439–445.

PIZZINI, A., BRIGNARDELLO, E., LEONARDI, L., DI MONACO, M. & BOCCUZZI, G. (1992). Aromatase fails to mediate the proliferative effects of adrenal androgens on cultured MCF-7 breast cancer cells. *Int. J. Oncol.*, **1**, 709–712.

POORTMAN, J., PRENEN, J., SCHWARZ, F. & THIJSSEN, J. (1975). Interaction of 5-en-androstene-3β,17β-diol with estradiol and dihydrotestosterone receptors in human myometrial and mammary cancer tissue. *J. Clin. Endocrinol. Metab.*, **40**, 373–379.

POULIN, R. & LABRIE, F. (1986). Stimulation of cell proliferation and estrogenic response by adrenal C19-delta5 steroids in the ZR-75-1 human breast cancer cell line. *Cancer Res.*, **46**, 4933–4937.

POULIN, R., BAKER, D. & LABRIE, F. (1988). Androgens inhibit basal and estrogen-induced cell proliferation in the ZR-75-1 human breast cancer cell line. *Breast Cancer Res. Treat.*, **12**, 213–225.

POULIN, R., SIMARD, J., LABRIE, C., PETITCLERC, L., DUMONT, M., LAGACE, L. & LABRIE, F. (1989). Down-regulation of estrogen receptors by androgens in the ZR-75-1 human breast cancer cell line. *Endocrinology*, **125**, 392–399.

ROCHEFORT, H. & GARCIA, M. (1984). The estrogenic and antiestrogenic activities of androgens in female target tissues. *Pharmacol. Ther.*, **23**, 193–216.

ROSENFIELD, R. & OTTO, P. (1972). Androstenediol levels in human peripheral plasma. *J. Clin. Endocrinol. Metab.*, **35**, 818-822.

SEGALOFF, A., HANKEY, B.F., CARTER, A.C., BUNDY, C. & MASNYK, J. (1980). Identification of breast cancer patients with high risk of early recurrence after radical mastectomy. III. Steroid hormones measured in urine. *Cancer*, **3**, 241-248.

SIMARD, J., LUTHY, I., GUAY, J., BELANGER, A. & LABRIE, F. (1986). Characteristics of interaction of the antiandrogen flutamide with the androgen receptor in various target tissues. *Mol. Cell. Endocrinol.*, **44**, 261-270.

THIJSSEN, J., POORTMAN, J. & SCHWARZ, F. (1975). Androgens in postmenopausal breast cancer: excretion, production and interaction with estrogens. *J. Steroid Biochem.*, **6**, 729-734.

WANG, D.Y., BULBROOK, R.D. & HAYWARD, J.L. (1975). Urinary and plasma androgens and their relation to familial risk of breast cancer. *Eur. J. Cancer*, **11**, 873-87.

ZAVA, D.T. & MCGUIRE, W.L. (1978). Human breast cancer: androgen action mediated by estrogen receptor. *Science*, **199**, 787-788.

ZUMOFF, B., LEVIN, J., ROSENFELD, R.S., MARKHAM, M., STRAIN, G.W. & FUKUSHIMA, D.K. (1981). Abnormal 24-hr mean plasma concentrations of dehydroisoandrosterone and dehydroisoandrosterone sulfate in women with primary operable breast cancer. *Cancer Res.*, **41**, 3360-3363.